# Comparison of the molecular characteristics of *Mycoplasma pneumoniae* from children across different regions of China

**DOI:** 10.1371/journal.pone.0198557

**Published:** 2018-08-23

**Authors:** Guanhua Xue, Ming Li, Na Wang, Jing Zhao, Bei Wang, Zhimin Ren, Chao Yan, Chengqing Wu, Yang Liu, He Sun, Min Xu, Hongmei Sun

**Affiliations:** 1 Department of Bacteriology, Capital Institute of Pediatrics, Chaoyang District, Beijing, China; 2 Department of Respiratory Medicine, Kunming Children’s Hospital, Kunming, China; 3 Institute of Antibiotics, Huashan Hospital, Fudan University, Shanghai, China; 4 Xinjiang Institute of Pediatrics, People’s Hospital of Xinjiang Uygur Autonomous Region,Xinjiang, China; 5 Department School of Public Health, Southeast University, Nanjing, China; 6 Department of Pediatrics, the Second Affiliated Hospital of Harbin Medical University, Harbin, China; Universidad Nacional de la Plata, ARGENTINA

## Abstract

Previous molecular characterization of *Mycoplasma pneumoniae* in China focused only on one or two cities. In this study, we characterized 835 samples from patients suspected to be infected with *M*. *pneumoniae*; these samples were collected in 2016 from pediatric patients from different regions of China. Multiple locus variable number tandem repeat analysis (MLVA), P1-restriction fragment length polymorphism (RFLP) analysis, and sequencing of the domain V of 23S rRNA were performed to compare genotype distribution across different locations. Two-hundred-and-thirteen samples tested positive for *M*. *pneumoniae* by PCR. P1 types were identified in 154 samples: 78.6% were type I and 21.4% were type II. Type I was the most prevalent genotype in five locations, except Nanjing where type II was the most common type (*p* < 0.01). Five distinct MLVA types were identified in the 172 samples. Genotype M4-5-7-2 was predominant at all locations, except Nanjing where type 3-5-6-2 was the most common (*p* < 0.01). Macrolide resistance-associated mutations were identified in 186 (76.3%) samples. The resistance rate differed with the location. This study showed that genotypes and macrolide resistance rate differed across China. The most prevalent genotype in China remains M4-5-7-2/P1-1. The resistance rate decreased, along with changes to the M4-5-7-2 genotype.

## Introduction

*Mycoplasma pneumoniae* is a common cause of respiratory infections, accounting for approximately 10–30% of all cases of community-acquired pneumonia (CAP); the prevalence increased to 50% in recent years [1]. The clinical presentation of *M*. *pneumoniae* infection widely varies, ranging from self-limiting to severe pneumonia, with extrapulmonary complications in children and adults [2].

This pathogen often spreads slowly through close contact in families, and the epidemic occurs every 3–7 years. The most recent world epidemic was first reported in Northern Europe, followed by some Asian countries [3–5]. Since the end of 2015, an increase has been noted in the incidence of these infections across Japan, China, and England [6–8], although incidence has remained stable in other countries. This scenario raises a question whether this is an endemic infection restricted to some local countries or a global epidemic. Extensive data collection and detailed studies are necessary to answer this question.

Macrolides are the first line of antibiotics used to treat *M*. *pneumoniae* infections. However, the resistance rate dramatically increased in the recent years. In Asia, over 90% of the isolates were resistant to macrolide [9]. In the United States, the macrolide-resistance rate was around 10% [10]. The rate varied from 1% to 30% in European countries [11]. Molecular characterization is the best way to understand the mechanisms underlying *M*. *pneumoniae* macrolide resistance. Certain multiple locus variable number tandem repeat analysis (MLVA) types are known to be associated with macrolide resistance [12], and the genotypes and the extent of macrolide resistance differ across countries as well as within a country [13, 14]. In China, the molecular characterization of *M*. *pneumoniae* has extensively focused on data from Beijing and Shanghai [15, 16]; data from other areas are scarce.

During the second half of 2015, we observed an increase in the prevalence of *M*. *pneumoniae* in Beijing [7]. To better understand the molecular characteristics of this endemic, we performed a multicentric study to compare the samples from five different areas (including six cities) in China in 2016.

## Materials and methods

### Ethics statement

This study was performed in compliance with the Helsinki Declaration (Ethical Principles for Medical Research Involving Human Subjects), and was approved by the Research Board of the Ethics Committee of the Capital Institute of Pediatrics, Beijing, China. All patient information was anonymized, and therefore, informed consent was not required, per the guidelines of the Ethics Committee of the Institute.

### Clinical specimens

**Fig 1:**
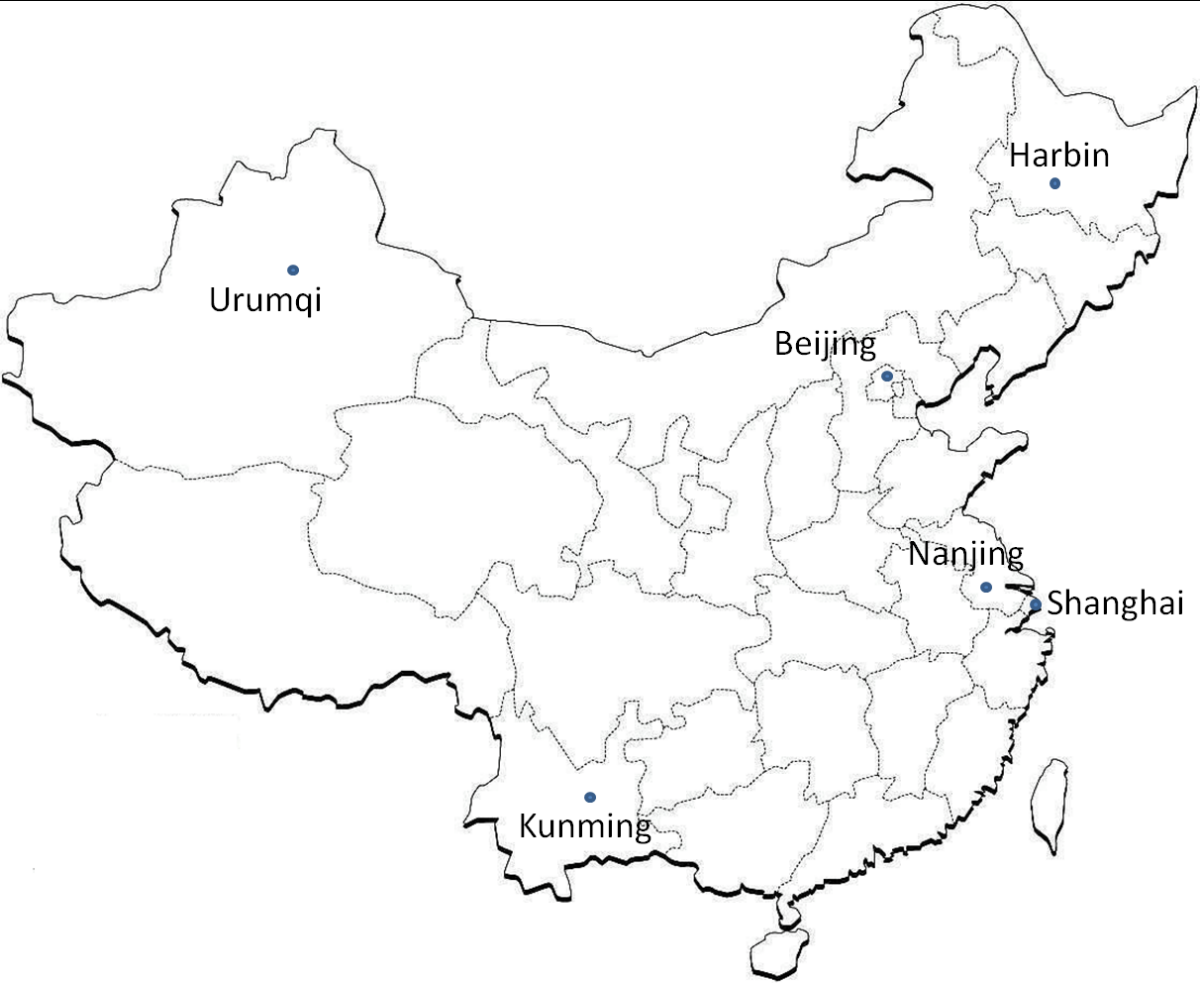
Locality map of different cities included in this study. A total of 835 samples from pediatric patients presenting with the symptoms of respiratory tract infection (RTI) belonging to five different geographical locations in China were collected from January to December 2016. Two-hundred-and-forty-three samples were from Beijing, which is located in mid-north China; 100, from Shanghai, located in the east; 275, from Kunming, Yunnan province, located in the south; 140, from Harbin, Heilongjiang province, located in the north; and 72, from Urumqi, Xinjiang province, located in the west. Ten *M*. *pneumoniae*-positive DNA samples isolated from 10 clinical specimens were collected from pediatric patients in Nanjing, which is close to Shanghai.

From January to December 2016, clinical respiratory specimens were collected from 835 pediatric patients presenting with symptoms of respiratory tract infection (RTI), belonging to five different geographical locations in China. Two-hundred-and-forty-three samples were obtained from Beijing, which is located in mid-north China (63 sputum, 45 oropharyngeal swab, 122 bronchoalveolar lavage, 12 pleural fluid, and 1 puncture fluid samples); 100, from Shanghai, which is located in the east (all nasopharyngeal aspirate samples); 275, from Kunming, Yunnan province, which is in the south (121 sputum and 154 BAL samples); 140, from Harbin, Heilongjiang province, which is located in the north (all oropharyngeal swab samples); and 72 from Urumqi, Xinjiang province, which is in the west (62 sputum and 10 BAL samples). Moreover, we obtained 10 *M*. *pneumoniae*-positive DNA samples (isolated from 10 oropharyngeal swabs) from different pediatric patients at different admission times in Nanjing, which is close to Shanghai (Fig 1). All specimens used in this study were part of routine patient management without any additional collection, sample types were different by site due to distinct study protocols. Most cases were clinically diagnosed as pneumonia, followed by bronchitis, asthma, and respiratory tract infections, associated with central nervous system infections. DNA was extracted from clinical samples using the QIAamp DNA Mini Kit (Qiagen, German), and *M*. *pneumoniae* was detected by real-time PCR [17].

### P1 gene typing

Nested PCR-restriction fragment length polymorphism analysis was used for P1 genotyping, as described previously [15], by directly using the DNA extracted from PCR-positive specimens. In brief, to detect the RepMP4 region of the P1 gene, the ADH1/ADH2 primer pair was used in the first PCR, and the ADH1in/ADH1M and ADH2in/ADH2M primer pairs were designed and used in the second PCR. To detect the RepMp2/3 region, the ADH3/ADH4 primer pair was used for the first PCR, and the ADH3in/ADH3M and ADH4in/ADH4M primer pairs were designed and used for the second PCR (S1 Table). The two nested RepMP4 PCR products were mixed together and digested using the restriction enzyme *Hae*III; the products were electrophoresed on 2% agarose gel (S1 Fig). The subtypes were tested by DNA sequencing and compared with the reference strain.

### MLVA typing

Multiplex PCR amplification-linked capillary electrophoresis of four loci (Mpn13, Mpn14, Mpn15, and Mpn16) was used for modified MLVA genotyping, and performed according to a previously described method [18, 19] and international guidelines [20].

### Detection of macrolide resistance

Mutations conferring macrolide resistance, including the common point mutations at positions 2063, 2064, 2611, and 2617 (according to *M*. *pneumoniae* numbering) within 23S rRNA gene, were tested as previously described [9].

### Statistical analysis

SPSS 21 package (SPSS Inc., Chicago, IL, USA) was used for statistical analysis. For comparison of proportions across different groups, the *χ*^2^ test was used. A *p* value of <0.05 was considered significant. For sites such as Nanjing that offered lesser samples, Fisher's exact test was used.

## Results

### Detection of *M*. *pneumoniae* from clinical specimens

Among 835 clinical samples, 213 (25.5%) tested positive for *M*. *pneumoniae* by real-time PCR including 72 (29.6%, 72/243) from Beijing; 28 (28%, 28/100) from Shanghai; 77 (28%, 77/ 275) from Kunming, Yunnan province; 20 (27.8%, 20/72) from Urumqi, Xinjiang province; and 16 (11.4%, 16/140) from Harbin, Heilongjiang province. The prevalence rate across these sites was similar, except in Harbin, where it was lower than other sites (*p* < 0.05).

### P1 genotyping

Due to the low levels of DNA in some samples, P1 genotyping was successful obtained from 154 *M*. *pneumoniae-*positive DNA samples. Type I (P1-1) was found in 78.6% (121/154), and type II (P1-2 and its variants P1-2a, P1-2c) in 21.4% (33/154) in these samples (Table 1). The proportion of type I and type II differs across cities: 89.1% and 10.9% in Beijing, 80% and 20% in Shanghai, 78% and 22% in Kunming, 83.3% and 16.7% in Harbin, 71.4% and 28.6% in Urumqi, and 20% and 80% in Nanjing. The distribution of the two types is similar in these cities, except in Nanjing, wherein type II is dominant (*p* < 0.01, Table 1).

**Table 1:** Molecular characteristics of *Mycoplasma pneumoniae* from different areas.

	Total	Beijing	Shanghai	Kunming, Yunnan	Urumqi, Xinjiang	Harbin, Heilongjiang	Nanjing
Specimens collected	N = 835	N = 243	N = 100	N = 275	N = 72	N = 140	-
*M*.*pneumoniae* -positive	213 (25.5%)	72 (29.6%)	28 (28%)	77 (28%)	20 (27.8%)	16 (11.4%)	10
P1 genotypes	N = 154	N = 55	N = 20	N = 50	N = 12	N = 7	N = 10
Type I (P1-1)	121 (78.6%)	49 (89.1%)	16 (80%)	39 (78%)	10 (83.3%)	5 (71.4%)	2 (20%)
Type II (P1-2)	6 (3.9%)	0	0	2 (4%)	1 (8.35%)	0	3 (30%)
Type II (P1-2a)^b^	1(0.65%)	0	0	1(2%)	0	0	0
Type II (P1-2c)^b^	26(16.9%)	6 (10.9%)	4 (20%)	8(16%)	1 (8.35%)	2 (28.6%)	5 (50%)
MLVA genotypes	N = 172	N = 59	N = 22	N = 56	N = 16	N = 9	N = 10
M4-5-7-2	124 (72.1%)	49 (83.1%)	17 (77.3%)	38 (67.9%)	12 (75%)	6 (66.7%)	2 (20%)
M3-5-6-2	38 (22.1%)	7 (11.9%)	5 (22.7%)	13(23.2%)	2 (12.5%)	3 (33.3%)	8 (80%)
M4-5-7-3	7 (4.1%)	2 (3.3%)	0	5 (8.9%)^a^	0	0	0
M4-5-5-2	2 (1.2%)	1 (1.7%)	0	0	1 (6.25%)	0	0
M3-6-6-2	1 (0.6%)	0	0	0	1 (6.25%)	0	0
Macrolide	N = 186	N = 60	N = 22	N = 70	N = 15	N = 9	N = 10
Resistance	142 (76.3%)	52 (86.7%)	18(81.8%)	52(74.3%)	12 (80%)	6 (66.7%)	2 (20%)
Sensitive	44 (23.7%)	8 (13.3%)	4 (18.2%)	18(25.7%)	3 (20%)	3 (33.3%)	8 (80%)

^a^Special repeat number of Locus Mpn15

^b^Type II variants were examined by DNA sequencing.

### MLVA genotyping

**Fig 2:**
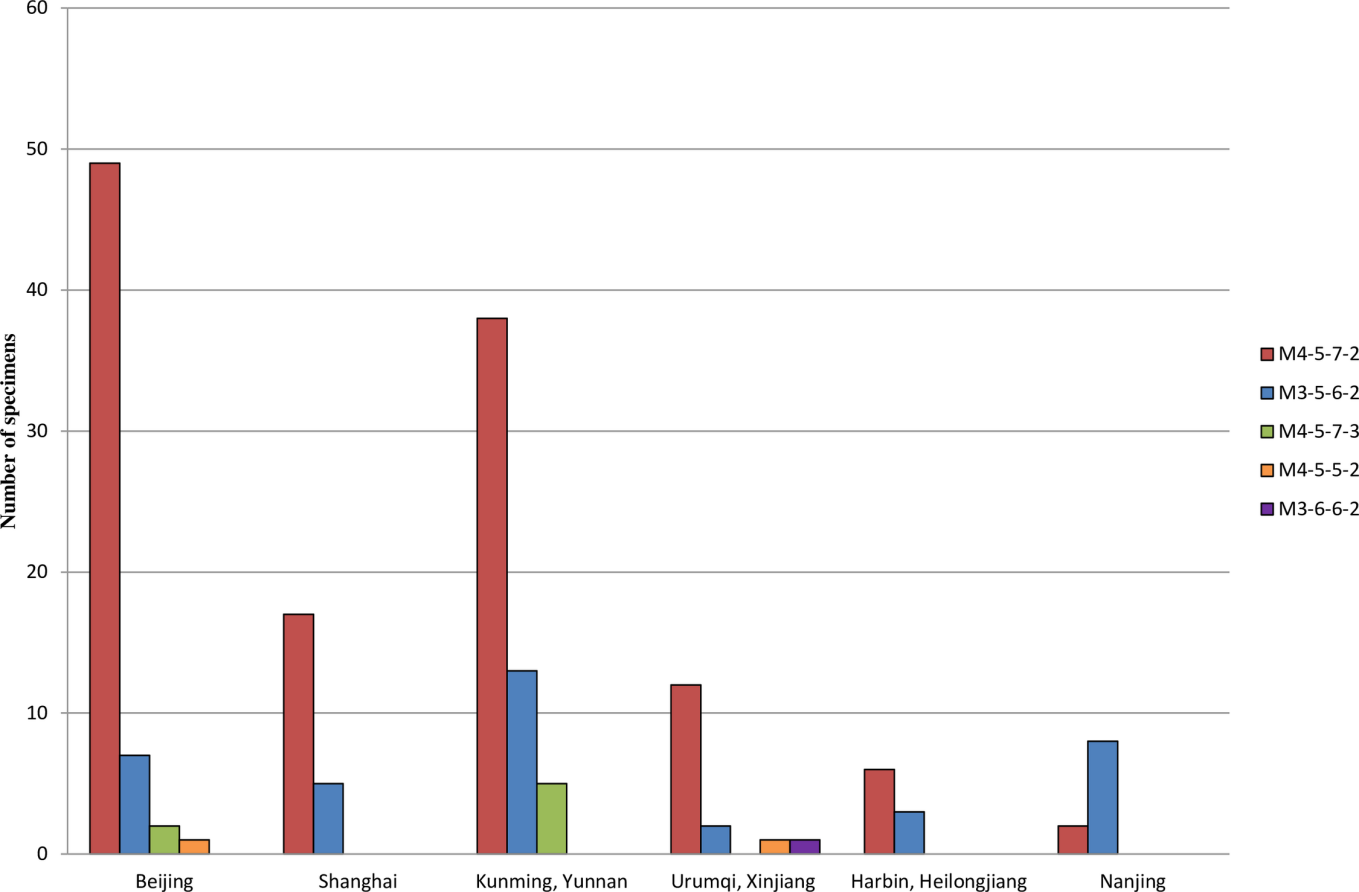
Genotype distribution across different sites. Different colors are used to represent the genotypes observed at different sites.

Due to the low levels of DNA, some specimens were failure to amplify all targets in MLVA. Full MLVA profile were obtained from 172 *M*. *pneumoniae* PCR-positive specimens and five distinct MLVA types were identified. The most common type was 4-5-7-2 (72.1%, 124/172), followed by type 3-5-6-2 (22.1%, 38/172), M4-5-7-3 (4.1%, 7/172), M4-5-5-2 (1.2%, 2/172), and M3-6-6-2 (0.6%, 1/172). Type M4-5-7-2 was predominant at all sites during the entire study duration, except in Nanjing where type 3-5-6-2 was most common (*p* < 0.01, Fig 2). Comparing the MLVA types with P1 genotype showed that the type M4-5-7-2 correlated with P1 Type I, and type M3-5-6-2 correlated with P1 Type II (*p* < 0.01, Table 1).

**Fig 3:**
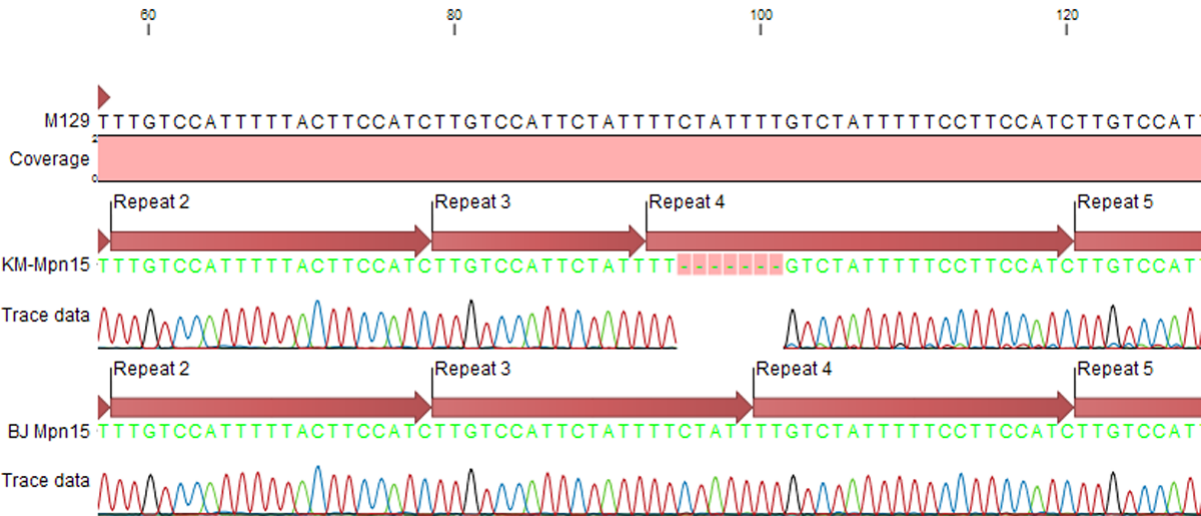
Sequence comparison of the locus Mpn 15. M129, *M*. *pneumoniae* reference strain; KM-Mpn15, the special sequence of Mpn15 from Kunming; BJ-Mpn15, the normal sequence of Mpn15 from Beijing; all repeat sequences in Mpn15 were 21-bp-long, except repeat 3, which was a 14-bp-long truncated sequence.

Two samples from Kunming showed a variation in MLVA types. Both samples were M4-5-7-3, with a size variation at the locus Mpn15. Sequencing showed that one of the repeats (repeat 3) in this locus was only 14-bp-long, compared to the other 21-bp-long samples (Fig 3). We then tested the presence of the highly variable locus Mpn1 in these two samples and found that both samples had the same copy number of 3. The two samples were collected from two different patients at different admission times. They might represent a new variation of MLVA types.

### Macrolide resistance gene mutations

**Fig 4:**
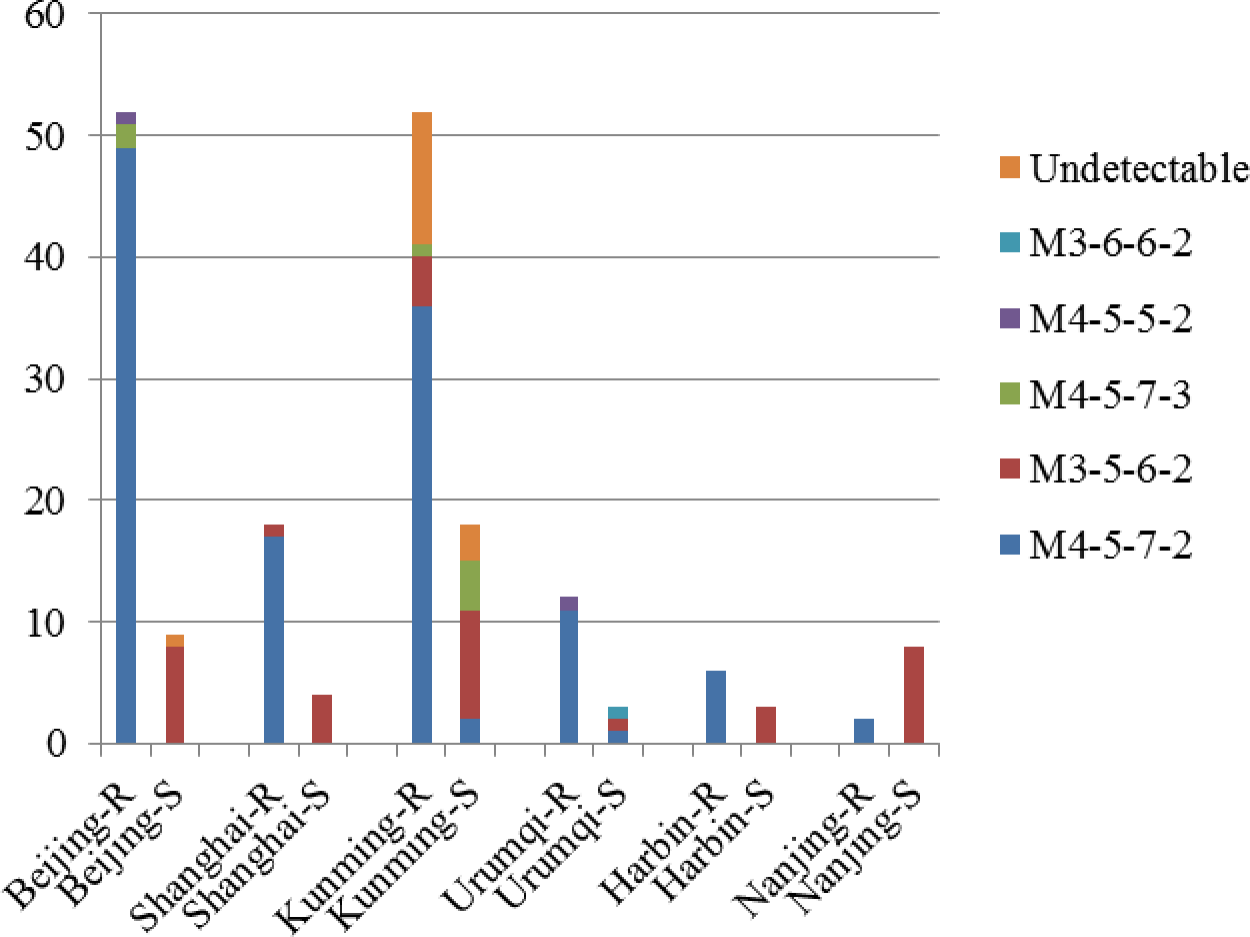
Different genotypes observed in macrolide-resistant and macrolide-sensitive specimens obtained from different sites in China. R, resistant; S, sensitive; Undetectable, the repeat number of the four loci could not be fully determined because of low DNA concentration.

Among the 223 *M*. *pneumoniae-*positive samples from six cities, 186 specimens were successfully tested for mutations in the 23S rRNA gene due to the low levels of DNA in some specimens. Of them, 142 (76.3%) had mutations associated with macrolide resistance, and 44 (23.7%) were wild type (Table 1). The resistance rates in different areas were as follows: Beijing, 86.7% (52/60); Shanghai, 81.8% (18/22); Kunming, 74.3% (52/70); Harbin, 66.7% (6/9); Urumqi, 80% (12/15); and Nanjing, 20% (2/10). The resistance rate in Nanjing was significantly lower compared to that at the other five sites (*p* < 0.01, Table 1). Sequencing analysis revealed the presence of the A2063G mutation in 137 (74.1%) specimens, and both A2063G and A2065C mutations were detected in a single specimen from Beijing. Three specimens had a mixture of A2063G and wild type, and one specimen had a mixture of A2064G and wild type. Of the 142 resistant specimens, 131 were genotyped successfully. The most prevalent MLVA type among resistant specimens was M4-5-7-2 (92.4%, 121/131), followed by M3-5-6-2 (3.8%, 5/131), M4-5-7-3 (2.3%, 3/131), and M4-5-5-2 (1.5%, 2/131) (Fig 4).

## Discussion

To better understand the recent endemic outbreaks as well as the molecular characteristics of *M*. *pneumoniae* strains found in China, we collected samples from six cities across the country in 2016. In China, the total detection rate was 25.51% in 2016, which is still higher compared to that in the previous non-epidemic years [21]. This indicated a new epidemic in some areas of China, which is consistent with our previous finding that the infection rate has been increasing from late 2015 [7]. Such a trend was observed in Japan and England in 2015 [6, 8] and in Central Slovenia in 2014 [22]. It is necessary to acquire more data from other sites to survey this trend. In this study, the detection rate at different locations was similar (>27%), except in the Heilongjiang province in northeast China (11.43%). The reasons underlying the low detection rate in this area are unclear. Sample types and transport methods might be two of the reasons, because all samples collected from Heilongjiang province were swabs, and the general consensus of several studies is that sputum is the best specimen for *M*. *pneumonia*, the other specimen types varied in different studies [23–25]. Also, as distinct study protocols used by site, the transport medium and methods were different. It can also affect the detection rate. Besides, colder climate can be another reason. In fact, a previous study showed that *M*. *pneumoniae* infection positively correlated with temperature, and that the infection rate of *M*. *pneumoniae* gradually increased with the increase in the minimum temperature [26].

P1-RFLP genotyping method has been applied for 20 years since its development [27, 28]. This method helps classify the organism into two major genotypes according the variations in the adhesin P1 gene type I and type II. In this study, the most prevalent genotype was type I (P1-1), which was in line with the findings in other countries [13, 4]. Although the proportion of P1-1 isolates varied from 71.4% to 89% in the five cities, except Nanjing, it was the dominant subtype. The samples from Nanjing were different in that type II (P1-2 and P1-2c) was the dominant subtype in those samples. Our previous studies in Beijing indicated that the P1 genotype started to switch from type I to type II as early as 2013 [29]. Since no historical data are available for Nanjing and because fewer samples were obtained from this site, even though these samples collected from different patients at different admission times, it is unclear whether this type II-dominant phenomenon is stable or dynamic in nature. Extensive data collection and detailed studies are necessary to answer this question.

Previous studies have established that the P1 type correlates with certain MLVA types [30]. In this study, MLVA 4-5-7-2 was found to be related to P1-1, and M3-5-6-2 to P1-2 and its variants. MLVA type M4-5-7-2 was the most prevalent type in our study, consistent with a previous study [4]. Unlike the other five cities, the most prevalent MLVA genotype in Nanjing was M3-5-6-2, corresponding to the predominant P1-2 and P1-2c subtypes. Differing geographical distribution of MLVA genotypes has also been observed in the USA [13]. Interestingly, we found two samples from Kunming, which showed a repeat length variation in the Mpn15 locus, where a 7-bp-long deletion (CTATTTT) was noticed in the third repeat sequence. This indicates that specific genotypes exist in certain regions. The biological significance of this variation is unknown.

The macrolide resistance rate is very high in China, reaching >90% annually [31, 9]. Interestingly, in this study, the average resistance rate was 76.3%. Considering single cities, the rate was decreased in Beijing and Shanghai [29, 32]. However, the specimens being compared belonged to different studies, and therefore, other factors such as the number of specimens tested and the method used among laboratories might affect this comparison. More studies are needed to verify the observed trends. Of all single cities, differences were observed in Nanjing compared to other sites, including a lower proportion of macrolide resistance (20%) and predominant genotype of M3-5-6-2. It was consistent with the study that the macrolide non-resistant rate of *M*. *pneumoniae* isolates with Mpn13-14-15-16 profile of 3-5-6-2 was significantly higher than that of other types. [33]. Genotype M4-5-7-2, which correlates with macrolide resistance, is predominant in other cities, and can be considered as the reason for the higher rate of resistance. These observations are consistent with those of the reports from China and the USA [34, 29]. The differences in the rate of macrolide resistance in Japan varied from 0% to 100% across four different cities [14]. However, no further studies have been undertaken to study the correlation with MLVA genotypes.

This study has several limitations. First, the number of samples obtained from some areas is quite low, with the least being 10 from Nanjing. This limits the power of statistical analysis as well as further examination of the specific characteristics of the samples obtained from Nanjing. Second, all specimens used in this study were part of routine patient management without any additional collection. As distinct study protocols used, sample types were different by site. The detection rate could be affected due to lack of systematic studies comparing this variable. Third, limited regions were included in this study. China is geographically diverse, and therefore, the microbiological characteristics of *M*. *pneumoniae* isolates obtained in this study might not completely represent the true status of all regions of China.

To summarize, we investigated the molecular characteristics of six cities across five different areas of China in 2016. The proportion of *M*. *pneumoniae* positive respiratory specimens was around 25.5%, and the most prevalent genotype was M4-5-7-2/P1-1 at all sites, except Nanjing. The macrolide resistant rate decreased, companied with decrease in the M4-5-7-2 genotype.

## Supporting information

S1 Table Primers used in P1-RFLP gene typing. (DOCX)

S1 Fig The electrophorese results of P1-RFLP typing. M: molecular weight marker, lane 1and 4: type II. lane 2, 3 and 5–8: type I.
(TIF)

## References

[1] Waites KB, Xiao L, Liu Y, Balish MF, Atkinson TP. *Mycoplasma pneumoniae* from the respiratory tract and beyond. Clin Microbiol Rev. 2017;30(3): 747–809. 10.1128/CMR.00114-16 28539503 PMC5475226

[2] Atkinson TP, Balish MF, Waites KB. Epidemiology, clinical manifestations, pathogenesis and laboratory detection of *Mycoplasma pneumoniae* infections. FEMS Microbiol Rev. 2008;32(6): 956–973. 10.1111/j.1574-6976.2008.00129.x 18754792

[3] Uldum SA, Bangsborg JM, Gahrn-Hansen B, Ljung R, Mølvadgaard M, Føns Petersen R, et al Epidemic of *Mycoplasma pneumoniae* infection in Denmark, 2010 and 2011. Euro Surveill. 2012;17(5): 20073 22321137 10.2807/ese.17.05.20073-en

[4] Whistler T, Sawatwong P, Diaz MH, Benitez AJ, Wolff BJ, Sapchookul P, et al Molecular characterization of *Mycoplasma pneumoniae* infections in two rural populations of Thailand from 2009 to 2012. J Clin Microbiol. 2017;55(7): 2222–2233. 10.1128/JCM.00350-17 28490485 PMC5483925

[5] Martínez MA, Ruiz M, Zunino E, Luchsinger V, Aguirre R, Avendaño LF. Identification of P1 types and variants of *Mycoplasma pneumoniae* during an epidemic in Chile. J Med Microbiol. 2010;59(Pt 8): 925–929. 10.1099/jmm.0.018333-0 20448063

[6] Yamazaki T, Kenri T. Epidemiology of *Mycoplasma pneumoniae* infections in Japan and therapeutic strategies for macrolide-resistant *M. pneumoniae*. Front Microbiol. 2016;7: 693 10.3389/fmicb.2016.00693 27242718 PMC4876131

[7] Yan C, Sun H, Zhao H. Latest surveillance data on *Mycoplasma pneumoniae* infections in children, suggesting a new epidemic occurring in Beijing. J Clin Microbiol. 2016;54(5): 1400–1401. 10.1128/JCM.00184-16 26912752 PMC4844719

[8] Brown RJ, Nguipdop-Djomo P, Zhao H, Stanford E, Spiller OB, Chalker VJ. *Mycoplasma pneumoniae* epidemiology in England and Wales: A national perspective. Front Microbiol. 2016;7: 157 10.3389/fmicb.2016.00157 26909073 PMC4754400

[9] Lin C, Li S, Sun H, Zhao H, Feng Y, Cao L, et al Nested PCR-linked capillary electrophoresis and single-strand conformation polymorphisms for detection of macrolide-resistant *Mycoplasma pneumoniae* in Beijing. J Clin Microbiol. 2010;48(12): 4567–4572. 10.1128/JCM.00400-10 20861333 PMC3008430

[10] Zheng X, Lee S, Selvarangan R, Qin X, Tang YW, Stiles J, et al Macrolide-resistant *Mycoplasma pneumoniae*, United States. Emerg Infect Dis. 2015;21(8): 1470–1472. 10.3201/eid2108.150273 26196107 PMC4517703

[11] Pereyre S, Goret J, Bébéar C. *Mycoplasma pneumoniae*: Current knowledge on macrolide resistance and treatment. Front Microbiol. 2016;7: 974 10.3389/fmicb.2016.00974 27446015 PMC4916212

[12] Ho PL, Law PY, Chan BW, Wong CW, To KK, Chiu SS, et al Emergence of macrolide-resistant *Mycoplasma pneumoniae* in Hong Kong is linked to increasing macrolide resistance in multilocus variable-number tandem-repeat analysis type 4-5-7-2. J Clin Microbiol. 2015;53(11): 3560–2564. 10.1128/JCM.01983-15 26338857 PMC4609705

[13] Diaz MH, Benitez AJ, Cross KE, Hicks LA, Kutty P, Bramley AM, et al Molecular detection and characterization of *Mycoplasma pneumoniae* among patients hospitalized with community-acquired pneumonia in the United States. Open Forum Infect Dis. 2015;2(3): ofv106 10.1093/ofid/ofv106 26284257 PMC4536330

[14] Ishiguro N, Koseki N, Kaiho M, Kikuta H, Togashi T, Oba K, et al Hokkaido Pediatric Respiratory Infection Study Group. Regional differences in prevalence of macrolide resistance among pediatric *Mycoplasma pneumoniae* infections in Hokkaido, Japan. Jpn J Infect Dis. 2016;69(3): 186–190. 10.7883/yoken.JJID.2015.054 26166502

[15] Sun H, Xue G, Yan C, Li S, Cao L, Yuan Y, et al Multiple-locus variable-number tandem-repeat analysis of *Mycoplasma pneumoniae* clinical specimens and proposal for amendment of MLVA nomenclature. PLoS One. 2013;8(5): e64607 10.1371/journal.pone.0064607 23737989 PMC3667773

[16] Liu Y, Ye X, Zhang H, Xu X, Wang M. Multiclonal origin of macrolide-resistant *Mycoplasma pneumoniae* isolates as determined by multilocus variable-number tandem-repeat analysis. J Clin Microbiol. 2012;50(8): 2793–2795. 10.1128/JCM.00678-12 22649013 PMC3421490

[17] Dumke R, Schurwanz N, Lenz M, Schuppler M, Lück C, Jacobs E. Sensitive detection of *Mycoplasma pneumoniae* in human respiratory tract samples by optimized real-time PCR approach. J Clin Microbiol. 2007;45(8): 2726–2730. 10.1128/JCM.00321-07 17537933 PMC1951254

[18] Dégrange S, Cazanave C, Charron A, Renaudin H, Bébéar C, Bébéar CM. Development of multiple-locus variable-number tandem-repeat analysis for molecular typing of *Mycoplasma pneumoniae*. J Clin Microbiol. 2009;47(4): 914–923. 10.1128/JCM.01935-08 19204097 PMC2668363

[19] Dumke R, Jacobs E. Culture-independent multi-locus variable-number tandem-repeat analysis (MLVA) of *Mycoplasma pneumoniae*. J Microbiol Methods. 2011;86(3): 393–396. 10.1016/j.mimet.2011.06.008 21704086

[20] Chalker VJ, Pereyre S, Dumke R, Winchell J, Khosla P, Sun H, et al International *Mycoplasma pneumoniae* typing study: interpretation of *M. pneumoniae* multilocus variable-number-tandem-repeat analysis. New Microbes New Infect. 2015;7: 37–40. 10.1016/j.nmni.2015.05.005 26236493 PMC4501435

[21] Zhao H, Li S, Cao L, Yuan Y, Xue G, Feng Y, et al Surveillance of *Mycoplasma pneumoniae* infection among children in Beijing from 2007 to 2012. Chin Med J (Engl). 2014;127(7): 1244–1248. 24709174

[22] Kogoj R, Mrvic T, Praprotnik M, Kese D. Prevalence, genotyping and macrolide resistance of *Mycoplasma pneumoniae* among isolates of patients with respiratory tract infections, Central Slovenia, 2006 to 2014. Euro Surveill. 2015;20: 37. 10.2807/1560-7917.ES.2015.20.37.30018 26536357

[23] Loens K, Van Heirstraeten L, Malhotra-Kumar S, Goossens H, Ieven M. Optimal sampling sites and methods for detection of pathogens possibly causing community-acquired lower respiratory tract infections. J Clin Microbiol. 2009;47(1): 21–31. 10.1128/JCM.02037-08 19020070 PMC2620840

[24] Reznikov M, Blackmore TK, Finlay-Jones JJ, Gordon DL. Comparison of nasopharyngeal aspirates and throat swab specimens in a polymerase chain reaction-based test for *Mycoplasma pneumoniae*. Eur J Clin Microbiol Infect Dis. 1995;14(1):58–61. 7729456 10.1007/BF02112622

[25] Xu D, Li S, Chen Z, Du L. Detection of *Mycoplasma pneumoniae* in different respiratory specimens. Eur J Pediatr. 2011;170(7):851–858. 10.1007/s00431-010-1360-y 21107602

[26] Tian DD, Jiang R, Chen XJ, Ye Q. Meteorological factors on the incidence of MP and RSV pneumonia in children. PLoS One. 2017; 12(3): e0173409 10.1371/journal.pone.0173409 28282391 PMC5345804

[27] Sasaki T, Kenri T, Okazaki N, Iseki M, Yamashita R, Shintani M, et al Epidemiological study of *Mycoplasma pneumoniae* infections in japan based on PCR-restriction fragment length polymorphism of the P1 cytadhesin gene. J Clin Microbiol. 1996; 34(2): 447–449. 8789036 10.1128/jcm.34.2.447-449.1996 PMC228818

[28] Cousin-Allery A, Charron A, de Barbeyrac B, Fremy G, Skov Jensen J, Renaudin H, et al Molecular typing of *Mycoplasma pneumoniae* strains by PCR-based methods and pulsed-field gel electrophoresis. Application to French and Danish isolates. Epidemiol Infect. 2000; 124(1): 103–111. 10722137 10.1017/s0950268899003313 PMC2810890

[29] Sun H, Xue G, Yan C, Li S, Zhao H, Feng Y, et al Changes in molecular characteristics of *Mycoplasma pneumoniae* in clinical specimens from children in Beijing between 2003 and 2015. PLoS One. 2017;12(1): e0170253 10.1371/journal.pone.0170253 28107399 PMC5249184

[30] Diaz MH, Benitez AJ, Winchell JM. Investigations of *Mycoplasma pneumoniae* infections in the United States: trends in molecular typing and macrolide resistance from 2006 to 2013. J Clin Microbiol. 2015;53(1): 124–130. 10.1128/JCM.02597-14 25355769 PMC4290910

[31] Zhou Z, Li X, Chen X, Luo F, Pan C, Zheng X, et al Macrolide-resistant *Mycoplasma pneumoniae* in adults in Zhejiang, China. Antimicrob Agents Chemother. 2015;59(2): 1048–1051. 10.1128/AAC.04308-14 25451048 PMC4335877

[32] Liu Y, Ye X, Zhang H, Xu X, Li W, Zhu D, et al Antimicrobial susceptibility of *Mycoplasma pneumoniae* isolates and molecular analysis of macrolide-resistant strains from Shanghai, China. Antimicrob Agents Chemother. 2009;53(5): 2160–2162. 10.1128/AAC.01684-08 19273684 PMC2681541

[33] Qu J, Yu X, Liu Y, Yin Y, Gu L, Cao B, et al Specific multilocus variable-number tandem-repeat analysis genotypes of *Mycoplasma pneumoniae* are associated with diseases severity and macrolide susceptibility. PLoS One. 2013;8(12): e82174 10.1371/journal.pone.0082174 24367502 PMC3867324

[34] Yan C, Sun H, Lee S, Selvarangan R, Qin X, Tang YW, et al Comparison of molecular characteristics of *Mycoplasma pneumoniae* specimens collected from the United States and China. J Clin Microbiol. 2015;53(12): 3891–3893. 10.1128/JCM.02468-15 26400785 PMC4652102

