# Supplementary material for: Comparison of the molecular characteristics of Mycoplasma pneumoniae from children across different regions of China
Source: PLoS One. 2018 Aug 23;13(8):e0198557. doi: 10.1371/journal.pone.0198557 (PMC6107135; doi:10.1371/journal.pone.0198557)
Supplement: S1 Table — (DOCX) [file pone.0198557.s001.docx]

Table S1. Primers used in P1-RFLP gene typing

| Primer | Sequence *(5’-3’) | Tm (°C) |
| --- | --- | --- |
| ADH1in | 180900 ATTCTCATCCTCACCGCCA 180918 | 65.68 |
| ADH1M | 182049 CCTTGGGATCCAAGTGATCA 182030 | 64.84 |
| ADH2in | 183090 CTGCTAACAATTCCGGATTGAG 183069 | 64.43 |
| ADH2M | 182030 TGATCACTTGGATCCCAAGG 182049 | 64.84 |
| ADH3in | 183130 TTGCTGCTAACGAGTACGAG 183149 | 60.76 |
| ADH3M | 184394 CAAATCCCACACACTCTCCA 184375 | 63.65 |
| ADH4in | 185623 TGACTGATACCTGTGCGG 185606 | 60.92 |
| ADH4M | 184375 TGGAGAGTGTGTGGGATTTG 184394 | 63.65 |

*Reference strain: U00089 (M129)
