# Supplementary figures and images for: Comparison of the molecular characteristics of Mycoplasma pneumoniae from children across different regions of China
Source: PLoS One. 2018 Aug 23;13(8):e0198557. doi: 10.1371/journal.pone.0198557 (PMC6107135; doi:10.1371/journal.pone.0198557)

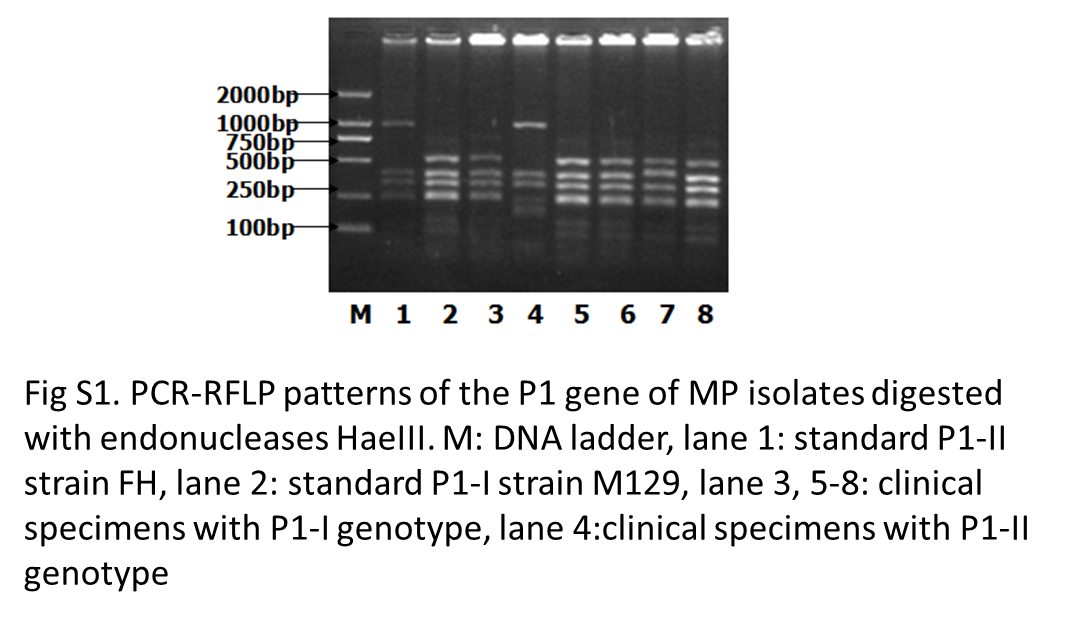

Supplement: S1 Fig — M: molecular weight marker, lane 1and 4: type II. lane 2, 3 and 5–8: type I. (TIF) [file pone.0198557.s002.tif]
